# In Vitro–In Vivo Correlation of Tianeptine Sodium Sustained-Release Dual-Layer Tablets

**DOI:** 10.3390/molecules27092828

**Published:** 2022-04-29

**Authors:** Ye-Ji Lee, Joo-Eun Kim

**Affiliations:** Department of Pharmaceutical Engineering, Catholic University of Daegu, Hayang-Ro 13-13, Hayang-Eup, Gyeongsan 38430, Korea; lyj7797@naver.com

**Keywords:** in vitro–in vivo correlation, in vitro dissolution study, in vivo pharmacokinetics study, tianeptine sodium, sustained-release tablet, daily administration

## Abstract

Tianeptine tablets are currently marketed to be designed for immediate-release tablets. The tianeptine has a short half-life, making it difficult to design for sustained-release tablets and achieve bioequivalence with the tianeptine immediate-release tablet (Stablon^®^). We established the in vitro–in vivo correlation (IVIVC) of three formulations of tianeptine sustained-release tablets according to their granule size. To evaluate sustained drug release, in vitro tests were performed in pH 1.2 media for 24 h. In vivo pharmacokinetic analysis was performed following oral administration of reference drug and test drug to beagle dogs. The dissolution profile revealed delayed release as the size of the granules increased. The dissolution results were confirmed in pharmacokinetic analysis, showing that the half-life was delayed as granule size increased. The final formulation and reference drug showed an equivalent area under the curve (AUC). Through this, IVIVC was established according to the size of the tianeptine sodium granules, which is the purpose of this study, and was used to predict in vivo pharmacokinetics from the formulation composition. This approach may be useful for determining optimal formulation compositions to achieve the desired pharmacokinetics when developing new formulations.

## 1. Introduction

According to the Food and Drug Administration, the in vitro–in vivo correlation (IVIVC) predicts an in vivo response to a drug based on the in vitro results of the orally administered drug [1]. Parameters that can be obtained in vitro include the rate of drug dissolution and amount of drug, whereas that which can be obtained in vivo is the amount of absorbed drug or blood concentration of the drug, such as the area under the concentration-time curve to infinity (AUC∞) and peak plasma concentration (Cmax) [2]. IVIVC is advantageous for evaluating changes in drug absorption in the body based on in vitro dissolution when the formulation is slightly changed [3]; it can also be used to predict the bioavailability (BA) and bioequivalence (BE) of drugs based on in vitro data [4]. In such cases, dissolution test results can be used to provide the desired information without the need for animal or human BE studies [5]. IVIVC is useful for predicting pharmacokinetics (PK); the ability to predict various outcomes can be used as an objective judgment criterion for developing new drugs [6]. This allows for the establishment of broader drug acceptance criteria and formulation stability and may be useful for predicting in vivo effects following changes to the manufacturing process, manufacturing site, or formulation components. Based on this method, drug efficacy can be predicted, particularly that of sustained-release double-layer tablets, promoting the efficient development of new drugs.

The tianeptine sodium dual-layer sustained-release (TSR) tablet is a selective serotonin reuptake enhancer used as an antidepressant developed through prior research. Animal studies confirmed that the response of the hypothalamus-pituitary-adrenal axis to stress was normalized during the continuous administration of tianeptine sodium (TS) [7]. In addition, TS can reduce the levels of glutamate, an excitatory neurotransmitter that causes tension and pain, to relieve depression and anxiety disorders [8,9]. Compared to other tricyclic antidepressants, TS has fewer anticholinergic side effects such as sedation, dry mouth, constipation, and adverse cardiovascular reactions, and thus is advantageous for treating the elderly and patients with alcoholism [10]. TS has a pKa of 4.22 and is mainly absorbed in vivo under acidic conditions [7]. When administered orally, TS shows an absolute bioavailability of approximately 99% and is completely absorbed, with approximately 1 h required (time to peak plasma concentration; Tmax) to reach the Cmax, indicating rapid absorption [11]. TS has a short half-life of approximately 2.5 h and a short duration of drug action. Therefore, the currently marketed tianeptine immediate-release tablets (Stablon^®^ tablet) are designed to be taken three times per day. For patients with depression who require long-term drug treatment, taking the drug several times per day lowers medication compliance and affects the treatment rate. Specifically, drug administration three times per day results in a 25% decrease in medication compliance compared to administration once per day [12]. However, tianeptine tablets that can be taken once per day have not been developed. Treatment failure often occurs because of the short half-life according to TS sustained-release studies. We hypothesized that the size of sustained-release granules affects BE, and examined the correlation between in vitro dissolution and in vivo PK studies according to the granule size [13].

In this study, we evaluated the IVIVC of the three TSR formulations with different particle sizes in the SR-layer granules and examined the bioequivalence with the reference drug and TSR tablets. During the research and development processes, when the granule size was small, the initial dissolution rate was fast compared to that of the reference drug, making it difficult to maintain the drug concentration for 24 h. Therefore, the formulation was optimized by increasing the granule size. To evaluate the bioequivalence of the TSR tablets and reference drug, we utilized the IVIVC to determine whether it can ensure the stability of TSR tablets and whether a tablet that is biologically equivalent to the reference drug can be developed [14]. Moreover, the TSR tablet developed in this study can be taken once per day, which may improve the long-term medication adherence of patients with depression.

## 2. Results and Discussion

### 2.1. Characterization according to the Manufacturing Process

**Figure 1:**
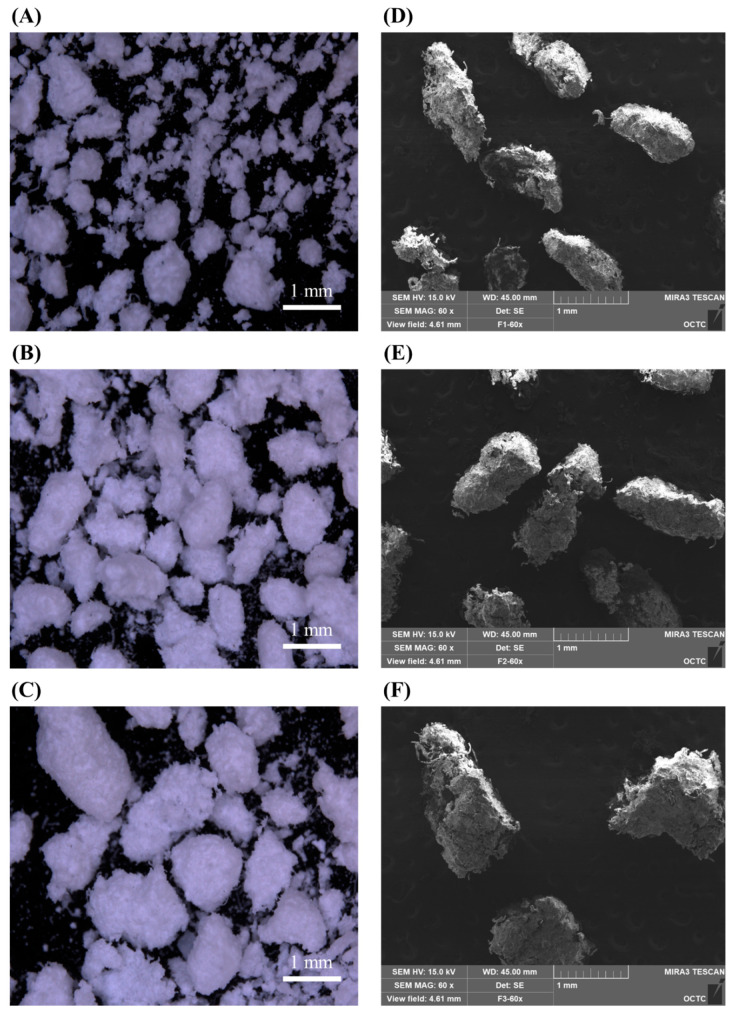
Morphology of (**A**) F1, (**B**) F2, (**C**) F3 particle observed through stereo binocular microscopy/(**D**) F1, (**E**) F2, (**F**) F3 particle observed through scanning electron microscope (scale bar = 1 mm).

Figure 1 is the particle size of the granules used for each formulation, confirmed with a stereo binocular microscope and scanning electron microscope. Formulation 1 has the smallest granular particle size in the formulation and formulation 3 has the biggest granular particle size in the formulation. The particle size of F1 is approximately 710 μm or less as granules can escape through a 24 mesh sieve, and the particle size of F2 is approximately 850 μm as granules can escape through a 20 mesh sieve. The particle size of F3 is confirmed to be less than approximately 1180 μm as granules that come out through a 16 mesh sieve. The difference in the in-process control result values was confirmed as an increase in the granule size (Table 1). In flowability measurements to investigate the characteristics of each formulation, the CI value decreased as the granule size increased. A smaller CI indicated better flowability. The final formulation, F3, showed the highest fluidity. After tableting, the hardness and tableting pressure were measured.

Consequently, the tensile strength was increased by reducing the granule size because of the increased bonding area available during direct compression. There was no significant change in tableting pressure or hardness, but it was confirmed that it decreased to some extent.

### 2.2. In Vitro Dissolution Studies

**Figure 2:**
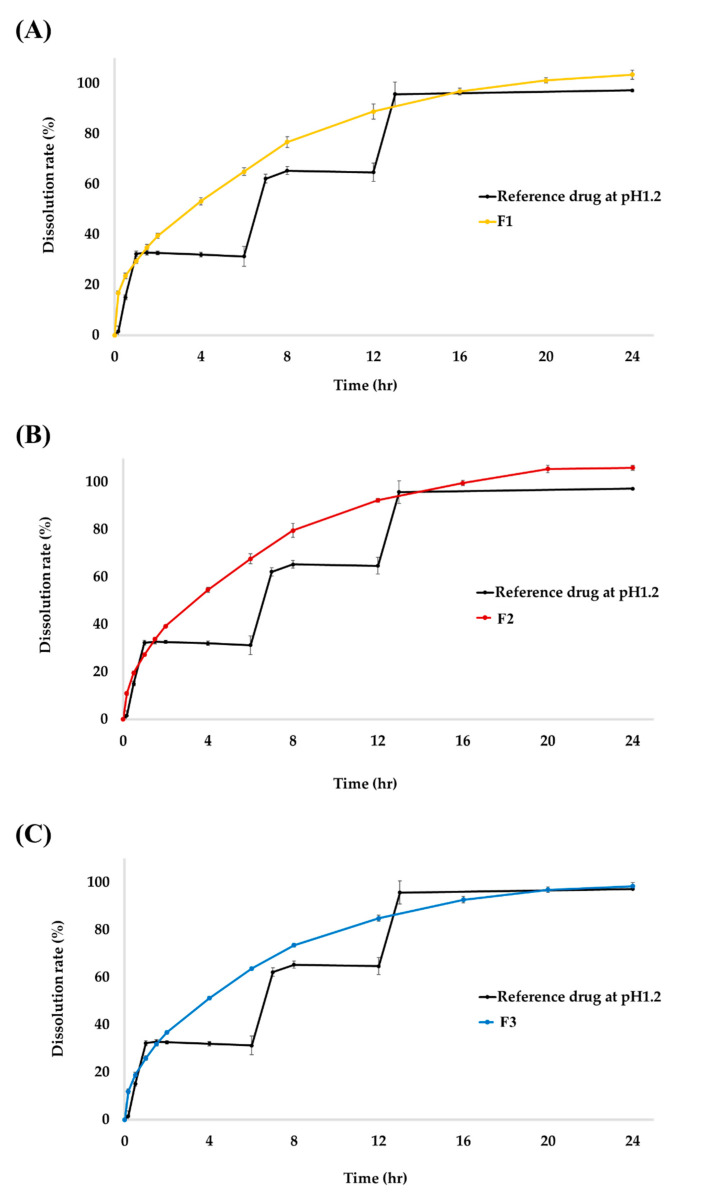
Dissolution profile of tianeptine sodium (TS) from reference drug and (**A**) F1, (**B**) F2, and (**C**) F3 test drug at pH 1.2. Each value represents the mean ± SD (*n* = 6). The reference drug, Stablon^®^ tablets (tianeptine sodium immediate-release tablet/taken three times per day); test drug, TSR tablets (tianeptine sodium sustained-release tablet/taken once per day).

The concentration (%) and sustained release pattern of TS according to three different SR granulate particle sizes were examined at pH 1.2 for 24 h (Figure 2). According to the paddle method of the USP<711>, the dissolution rate standards for SR tablets should be 20–30%, approximately 50%, and 80% or more at the 1 h, 4 h, and 12 h, respectively [15]. At pH 1.2, the dissolution rates of TSR tablets (F1) were 29.4% at 1 h, 53.2% at 4 h, and 88.8% at 12 h. For F2, the dissolution rate was 27.2% at 1 h, 54.6% at 4 h, and 92.3% at 12 h. The dissolution rate of the final formulation F3, the dissolution rate was 25.9% at 1 h, 51.2% at 4 h, and 84.9% at 12 h. Thus, the initial dissolution rate was proportionally delayed as the ratio of the SR granulate particle size increased. Additionally, all tablets achieved a final dissolution rate of 100% and showed a dissolution graph that increased over time and was maintained after an intermediate time point.

### 2.3. In Vivo Pharmacokinetic Studies

**Figure 3:**
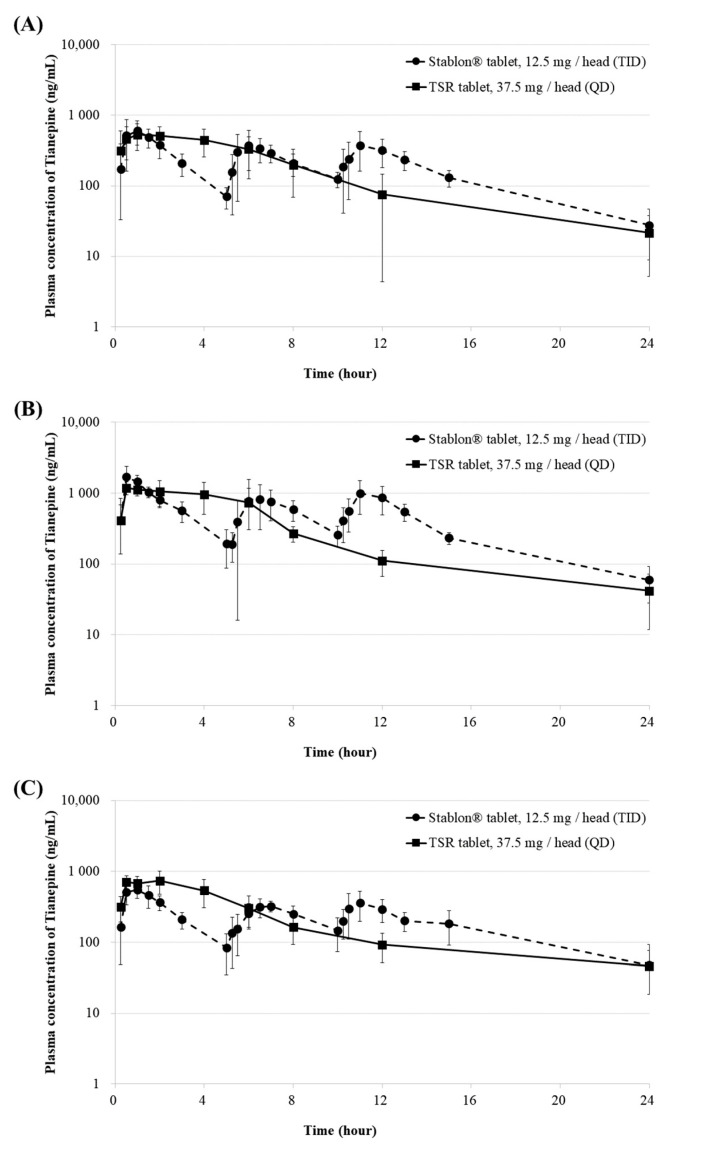
Plasma concentration-time profiles of (**A**) F1 (**B**) F2, and (**C**) F3 of TSR tablets (37.5 mg/head [QD]) and Stablon^®^ tablets (12.5 mg/head [TID]). Each value represents the mean ± SD (*n* = 6). Stablon^®^ tablets, tianeptine sodium immediate-release tablets taken three times per day; TSR tablets, tianeptine sodium sustained-release tablets taken once per day.

We performed PK studies a total of 3 times according to formulations with different granule sizes. This study randomly included 12 healthy beagle dogs for each PK study. In addition, for each PK study we performed a total 12 beagle dogs were used, 6 for the reference drug and 6 for the test drugs. Comparison of the PK of tianeptine following oral administration of the reference drug (12.5 mg) three times per day to 6 beagle dogs and test drug (37.5 mg) once per day to 6 beagle dogs showed typical sustained-release profile, such as a delay in the Tmax and a long half-life. As shown in Table 2 and Figure 3, the PK evaluation and PK parameters according to each prescription are presented.

The Tmax of the primary PK study of the F1 tablet with the smallest granular particle size (using a 24-mesh sieve) and reference drug were 0.25–4 h and 0.5–6 h. The Cmax of F1 was 645.02 ± 190.15 ng/mL at 2.0 (0.25–4) h, and AUClast of F1 was 4284.49 ± 1338.96 ng·h/mL. The Cmax and AUClast of the reference drug were 745.25 ± 238.75 ng/mL and 4559.86 ± 1064.77 ng·h/mL, respectively. The Tmax of the F2 tablet and reference drug were 0.5–4 h and 0.5–11 h. Thus, granular particles with a smaller size appeared to dissolve more rapidly compared to those with a larger size. The equivalence of Cmax and AUClast was not confirmed, even based on the results of the second PK study. The AUClasts of the F3 tablet and reference drug were 5130.3 ± 714.9 and 4801.5 ± 923.8 ng·h/mL, respectively. The Cmax values of the F3 tablet and reference drug were 823.8 ± 217.2 and 620.8 ± 111.4 ng/mL, respectively. The Cmax and AUClast of the F3 tablet and reference drug did not significantly differ (Mann-Whitney test, *p* > 0.05) [16]. Moreover, the terminal half-life gradually increased to 4.88, 5.5, and 7.6 h in that order. Therefore, the initial dissolution rate could be controlled by decreasing the surface area as the particle size increased, and the drug concentration was maintained for 24 h through AUClast equivalence. Based on these results, TSR F3 tablets may improve medication compliance with TS antidepressants, which currently must be taken three times per day.

However, the means AUClast for the reference drug are 4560, 10,607 and 4802, respectively for F1, F2 and F3. Each PK parameter for the reference drug among the 3 formulations is not clear and not constant. We have used a proper statistical test or some sort of transformation to compare the values of the test drugs among the formulations to adjust for these variable references. So, we have used a proper statistical test such as geometric mean ratio (Test/Ref, %) and point estimation (90% CI (Lower to upper)). As shown in Table 2, the geometric mean ratio of F1, F2, and F3 does not differ sequentially depending on the granular particles, but the geometric mean ratio of F3 seems to represent the most statistically close to bioequivalence value. So, we have compared theoretically to allow an appropriate comparison between the 3 formulations for all reference drug values.

### 2.4. IVIVC

We confirmed the IVIVC correlation through in vitro dissolution and in vivo PK studies of TSR tablets and Stablon^®^ tablets. In in vitro and in vivo analysis, Stablon^®^ tablets, as the reference drug, were administered three times at 6-h intervals, whereas TSR tablets were administered once. The dissolution rate of the F1 formulation, which had the smallest granule particle size, was similar to that of the reference drug at 1 h but increased compared to that of the reference drug at 12 h. However, the results of in vivo PK analysis confirmed that the F1 formulation did not last as long as the plasma drug concentration of the reference drug after 12 h, although the plasma drug concentration increased after initial of 2 h administration. F1 formulation with a small granule particle size was quickly absorbed at the beginning, but washed out in the body after a 12 h period indicating a lower AUC compared to the reference drug.

The particle size of the F2 formulation was larger than that of the F1 formulation. The F2 formulation also showed a lower dissolution rate and blood drug concentration compared to those of the reference drug. This result indicates that the F1 and F2 formulations were not bioequivalent to the reference drug. However, the F3 formulation, which had the largest granule particle size, showed the same dissolution rate as the reference drug at the 1 h and 12 h dissolution rate. PK analysis in beagles showed that the mean Cmax and AUClast showed bioequivalence for the F3 formulation and the control drug. The F3 formulation, which was biologically equivalent to the reference drug, was selected as the final formulation.

We did not clearly believe in the results of particle size approaching bioequivalence. In the preliminary test, it was found that the size of granules had an effect on the sustained-release tablet of tianeptine, but there was no clear evidence. So, this study was conducted. The only difference between the three PK data of the reference drug and the test drug was the size of the granules. We used the same amount of active ingredient and the same excipient. At this time, the difference that presented a result close to bioequivalence in the comparison of the PK results between the reference drug and the test drug is the point where only the size of the granules by the mesh was changed among the process parameters.

As shown in Figure 1, the size of the granules is clearly different due to the mesh sieve. Also, as shown in Figure 2, the dissolution profile result also slows down depending on the size of the granules. As shown in Figure 3, it can be seen that only the PK result of F3 approaches a value suitable for bioequivalence.

IVIVC can be used to predict the results of in vivo studies following slight changes to the manufacturing process, manufacturing site, or formulation components used to produce controlled-release pharmaceuticals. Finally, we established IVIVC for developing controlled-release TSR tablets, which may enable the manufacture of controlled-release drugs.

We confirmed that the sustained-release dissolution pattern differed depending on the granule size, which affected the PK pattern. PK analysis in beagles showed that only the granules with sizes that passed through 16-mesh during the manufacturing process showed bioequivalent results for Cmax and AUC compared to the reference drugs. Based on these results, we established an IVIVC and developed TSR tablets that are bioequivalent to the reference drug.

## 3. Materials and Methods

### 3.1. Materials

Tianeptine sodium was obtained from Hanseochem (Pyeongtaek, Korea). The reference drug was provided by Korea Pharma (Seoul, Korea). Hydroxypropyl methylcellulose (HPMC) and polyethylene oxide (PEO) were purchased from Colorcon, Inc., (Harleysville, PA, USA). Microcrystalline cellulose was purchased from JRS Pharma Co., Ltd. (Patterson, NY, USA). d-Mannitol was purchased from ROQUETTE Ltd. (Lestrem, France). Magnesium stearate was purchased from Nitika Pharmaceutical Specialties Pvt., Ltd. (Nagpur, India). Colloidal silicon dioxide (Aerosil 200) was purchased from EVONIK Health Care Co., Ltd. (Rellinghauser Strasze, Essen, Germany).

Pentagastrin was provided by Sigma-Aldrich (St. Louis, MO, USA) and the Nonclinical Research Center, QuBEST BIO Co., Ltd. (Yongin, Korea). The *N*,*N*-dimethylformamide and 0.9% saline solution required to manufacture pentagastrin were purchased from Duksan General Science and Dai Han Pharm. Co., Ltd. (Seoul, Korea). All other chemicals and solvents were of analytical grade and obtained commercially.

### 3.2. Preparation of TSR Tablets

**Figure 4:**
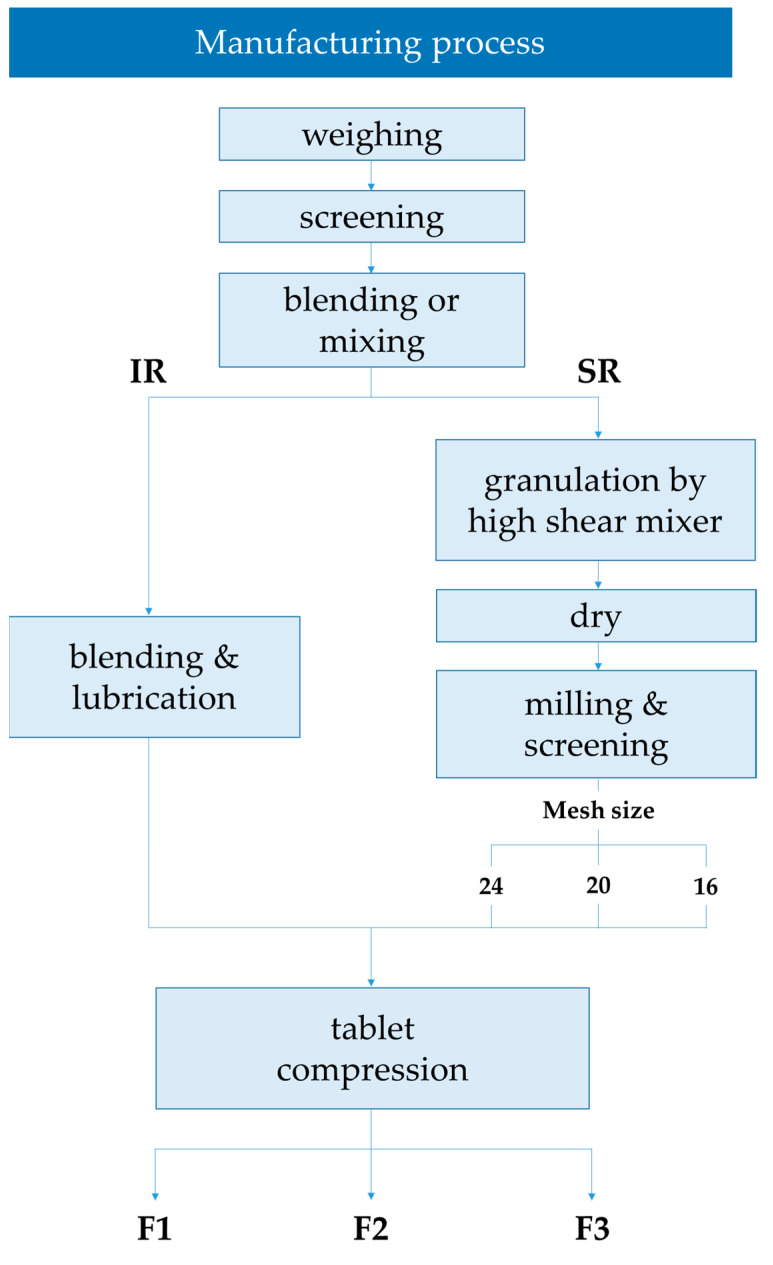
Manufacturing process of the TSR Tablet.

As shown in Figure 4, TSR dual-layer tablets consist of IR (immediate-release) and SR (sustained-release) layers. Three types of tablets were prepared by varying the granulation size of the SR layer. We controlled the size of granulation to investigate its effect on drug release from the tablet. The IR layer was manufactured via simple mixing, and the SR layer was manufactured via wet granulation using a high-shear mixer. TS, microcrystalline cellulose, D-mannitol, magnesium stearate, and colloidal silicon dioxide were used to prepare the IR formulation, whereas TS, HPMC, PEO, D-mannitol, magnesium stearate, and colloidal silicon dioxide were used to prepare the SR formulation. The compositions of the TS IR and SR tablets are listed in Table 3. To manufacture the SR layer, D-mannitol and colloidal silicon dioxide were screened through 40-mesh, and then TS, HPMC, and PEO were mixed. Using a high-shear mixer, deionized water (DIW) was added as a binder to prepare the granules, which were dried in an oven at 60 °C until the moisture content was less than 2.0%. The dried granules were screened through a mesh sieve and mixed with magnesium stearate as the lubricant. A larger mesh sieve number allowed smaller particles to pass through the sieve. The SR granules of F1 were screened with 24-mesh, F2 with 20-mesh, and F3 with 16-mesh. Therefore, the SR particle sizes of the three formulations increased. The resulting SR granules and IR were compressed by a 10 kN compaction force using a tablet compression machine (PR-LD22, PTK, Gimpo, Korea) to prepare the final TSR dual-layer tablets.

### 3.3. Characterization according to the Manufacturing Process

We investigated the effect of the tablet properties on the granule particle size of the SR layer [17]. A stereo binocular microscope (SMZ18 Contact Scope, Nikon, Tokyo, Japan) and scanning electron microscope (SEM, MIRA3LMH, Tescan, Brno-Kohoutovice, Czech Republic) were used to confirm the differences in particle size of the granules used for each formulation. The flowability of the mixture of each formulation prepared using the three manufacturing methods was calculated using Carr’s index (CI) [18]. The bulk and tap densities were measured using a graduated measuring cylinder. The bulk density is defined as the mass of many particles in the powder sample divided by their total occupied volume. The tap density was obtained after mechanically tapping a graduated measuring cylinder containing the powder sample. The flowability formula is shown below. A CI value of ≥25 indicates poor flowability, whereas a CI value of ≤15 indicates good flowability.

$$Carr^{\prime }s\;Index=\frac{\mathrm{Tap}\;\mathrm{density}-\mathrm{Bulk}\;\mathrm{density}}{\mathrm{Tap}\;\mathrm{density}}$$

An in-process control was performed for each prepared tablet. Hardness was measured using a Screw Test Stand (ALX-J, Wenzhou Tripod Instrument Manufacturing Co., Ltd., Wenzhou, China) in units of kilopond (kp). The tableting pressure was confirmed by measuring the tableting pressure of the tableting machine in units of kilonewtons.

### 3.4. In Vitro Dissolution Studies

In vitro dissolution studies were performed according to USP <711> Dissolution Apparatus 1 (Basket Apparatus) with six tablets of TSR tablets and six tablets of reference drugs (Stablon^®^) [15]. Stablon^®^ tablets were administered at 1T every 0, 6, and 12 h after the start of dissolution to reproduce the dose three times per day, and dissolution solutions were additionally collected at 7 and 13 h later. The dissolution test was performed using a PTWS 120D (Pharma Test Apparatebau AG, Siemensstr, Hainburg, Germany) in triplicate at 37.0 ± 0.5 °C and a basket speed of 100 rpm. The test medium was 0.1 N hydrochloric acid solution (pH 1.2). Medium samples (4 mL) were drawn at 0.17, 0.5, 1, 1.5, 2, 4, 6, 8, 12, 16, 20, and 24 h. The collected samples were filtered through a 0.45-μm syringe RC filter. The filtered dissolution sample (10 µL) was analyzed using HPLC (Breeze QS HPLC, UV/Vis 2489; Waters Corporation, Milford, MA, USA) at a UV detector wavelength of 220 nm. TS was separated under mobile phase isocratic conditions consisting of potassium dihydrogen phosphate in water and acetonitrile (65:35, *v*/*v*) using an XBridge^®^ C18 col-umn (4.6 mm × 15 cm, 5.0 µm). The flow rate was 1 mL/min and the total run time was 10.0 min.

### 3.5. In Vivo Pharmacokinetic Studies

#### 3.5.1. Animals

PK studies were conducted in male Covance beagle dogs (8.7–10.8 kg, 11–20 months old, Orient Bio, Inc., Sungnam, Korea). The beagle dog is a widely used species in pre-clinical studies and was used in this study because abundant basic data is available for PK studies. The minimum number of beagle dogs used was used to evaluate the PK of the test compounds. All experiments were performed at QuBEST Bio Co., Ltd. (Yongin, Korea). (Nonclinical Research Center). The animal study protocol complied with the institutional guidelines of the International Animal Care and Use Committee. (IACUC No.: KPC-IACUC-P203051, KPC-IACUC-P203039, and KPC-IACUC-P2030021) [19].

#### 3.5.2. Drug Administration and Blood Sampling

Beagle dogs were randomly divided into two equal groups, each containing six males. Beagle dogs were fasted for 12 h prior to drug administration and 4 h post-administration. At 30 min before drug administration, pentagastrin (Sigma-Aldrich), stored frozen at −20 °C, was intramuscularly administered to the hind legs of beagle dogs based on the weight of each individual (0.25 mL/kg). The reference drug (Stablon^®^ tablets 12.5 mg three times) and test drug (TSR tablet 37.5 mg once) were administered, and one tablet of the reference drug was administered three times per day at 0, 5, and 10 h. The test drug was administered once per day at one tablet at 0 h. After drug administration, 25 mL of water was provided to each animal. Blood samples (500 µL) were collected in heparinized tubes at 0, 0.25, 0.5, 1, 1.5, 2, 3, 5, 5.25, 5.5, 6, 6.5, 7, 8, 10, 10.25, 10.5, 11, 12, 13, 15, and 24 h after administration of the reference drug and at 0, 0.25, 0.5, 1, 2, 4, 6, 8, 12, and 24 h after administration of the F1, F2, and F3 test tablets.)

#### 3.5.3. Sample Analysis

The collected blood was centrifuged at 4000 rpm at 4 °C for 10 min to collect plasma samples, which were stored at below −70 °C until analysis. All frozen plasma samples were thawed at 20 °C prior to analysis. The concentration of TS in the plasma was analyzed using ultra-high-performance liquid chromatography-tandem mass spectrometry (Agilent 1290 Infinity II, Agilent Technologies, Santa Clara, CA, USA; QTRAP^®^ 4500 System, AB SCIEX, Framingham, MA, USA). A 2-µL sample was injected into the C18 column (4.6 × 50 mm, 1.8 μm). The mobile phase consisted of acetonitrile and 0.1% formic acid, provided as isocratic elution at a flow rate of 0.3 mL/min. The total run time was 3 min.

#### 3.5.4. Statistical Analysis

Non-compartmental PK modeling of the time-versus-plasma concentration profiles of each beagle dog after intramuscular administration was performed. The half-life (t_1/2_), AUC, and clearance (Cl) of absorption and clearance for PK analysis were non-compartmental based on analysis with Phoenix^®^ WinNonlin^®^ software (v. 8.2, Certara, Princeton, NJ, USA). When calculating the average concentration and PK parameters, the concentration below the lowest limit of quantification was marked as below the limit of quantification and regarded as “0”. Statistical significance was set at *p* ≤ 0.05. The Cmax and time to peak plasma concentration (Tmax) were obtained from the fitted concentration-time curve based on the compartment model. The bioavailability of TS was calculated using a standard equation (Toutain & Bousquet-Melou, 2004). PK parameters were set as the mean ± standard deviation (SD) values. The PK parameters were the AUC from time zero to infinity, AUC from time zero to the last quantifiable concentration (AUClast), and Cmax. Bioequivalence for the PK parameters was determined using analysis of variance if the 90% confidence intervals for the ratio of geometric least-squares means of the treatments compared were completely contained within the pre-defined equivalence margin of 0.8–1.25. Other main effects were examined at the 5% significance level for residual error (signify square error) using the analysis of variance model as the error term.

### 3.6. IVIVC

IVIVC was used to derive the interrelationship of mechanisms that significantly affect in vitro dissolution evaluation and in vivo PK evaluation based on changes occurring during formulation design and was used for drug development. We examined whether meaningful results could be derived from in vitro and in vivo studies of the three formulations by controlling the particle size of the granules during the formulation design process. In addition, for orally administered drugs, the correlation between IVIVC and high-permeability drugs or controlled-release drugs can be predicted using the biopharmaceutical classification system (BCS) [20]. BCS class I with high permeability and class II can confirm or predict IVIVC, whereas class III and IV have little or no ability to predict IVIVC. TS is a BCS class I drug with high solubility and permeability; therefore, the IVIVC correlation could be evaluated [21]. Three formulations of TS with different granule sizes were prepared, and in vitro dissolution and in vivo PK data were used to estimate the in vivo dissolution profile through IVIVC according to Food and Drug Administration guidelines [1].

## 4. Conclusions

Stablon^®^ tablet is a tianeptine immediate-release tablet; in contrast, the tianeptine SR tablet has a short half-life, making it difficult to achieve bioequivalence with the Stablon^®^ tablet, and there are currently no products on the market. In this study, we attempted to confirm the IVIVC of three tianeptine bilayer sustained-release tablets (TSR tablets) with different granular particle sizes. The TSR tablet was developed as a new formulation of TS that can be administered once per day, improving the convenience for patients with depression. The developed TSR tablets showed the same effect as Stablon^®^ antidepressants, which should be administered three times per day. A dual-layer SR tablet with both IR and SR layers was manufactured by using a polymer matrix. The dissolution rate, sustained-release pattern, and gastric retention potential of the prepared TSR tablets were evaluated, and a PK study was conducted; the results with those of the reference drug. When the size of the granules was small during the research and development process, the initial dissolution rate was higher than that of the reference drug, making it difficult to maintain the drug concentration for 24 h. Therefore, the formulation was optimized by increasing the granule size, which slowed dissolution.

The optimized TSR tablets were composed of TS, microcrystalline cellulose, D-mannitol, HPMC, PEO, colloidal silicon dioxide, and magnesium stearate. To effectively control drug release, the SR layer was prepared with wet granules using a high-shear mixer and sieved in the order of 24, 20, and 16 mesh. In the three dissolution tests, all formulations showed a dissolution rate of 100% and sustained release pattern at pH 1.2, and the particles floated. However, the early dissolution rate decreased slightly as the granule size increased. As the granule size of the TSR formulation decreased, the early dissolution rate was too fast for the reference drug; therefore, the drug concentration of F1, F2 could not be maintained for 24 h. Therefore, for bioequivalence, increasing the granule size led to a dissolution profile that exhibited delayed release. The TSR tablet was composed of a dual-layer tablet with an immediate-release (IR) layer and sustained-release (SR) layer to determine its bioequivalence with a reference drug based on the controlled release. The IR layer contained a small amount of TS, leading the Cmax to be equivalent to that of the reference drug. In the SR layer, granules composed of a hydrophilic polymer matrix, such as hydroxypropyl methylcellulose (HPMC) and polyethylene oxide (PEO), are used to maintain the drug concentration in the stomach for 24 h. We observed a correlation between the in vitro and in vivo results according to the TS granule size. Through IVIVC, the in vitro dissolution pattern and in vivo PK can be predicted during drug development, enabling the production of controlled-release formulations. Our results indicate that developing a drug with an equivalent Cmax compared to the existing drug with fewer administrations will increase patient compliance, leading to improved effects in patients with depression who require long-term treatment.

## Figures and Tables

**Table 1:** Characterization according to the manufacturing process.

Characterization	SR Formulation		
	F1	F2	F3
Mesh size	24	20	16
Bulk density	0.43 ± 0.01	0.39 ± 0.01	0.34 ± 0.02
Tap density	0.57 ± 0.02	0.50 ± 0.02	0.42 ± 0.01
Carr’s Index (%)	23.4 ± 1.60	22.2 ± 2.10	19.5 ± 1.46
Hardness (kp)	16.1 ± 0.01	15.7 ± 0.01	15.0 ± 0.02
Tableting pressure (kN)	18.2 ± 0.02	17.4 ± 0.01	16.5 ± 0.01

**Table 2:** PK study results of reference drug and test drug, pharmacokinetic parameters.

PK Parameter *	F1		F2		F3	
	Reference Drug (12.5 mg)	Test Drug (37.5 mg)	Reference Drug (12.5 mg)	Test Drug (37.5 mg)	Reference Drug (12.5 mg)	Test Drug (37.5 mg)
C_max_ (ng/mL)	745.25 ± 238.75	645.02 ± 190.15	1796.0 ± 592.7	1310.8 ± 213.1	620.8 ± 111.4	823.8 ± 217.2
T_max_ (h) **	1.0 [0.5–6]	2.0 [0.25–4]	0.5 [0.5–11]	1.5 [0.5–4]	0.8 [0.5–1]	2.0 [1–4]
AUC_last_ (ng·h/mL)	4559.86 ± 1064.77	4284.49 ± 1338.96	10607.6 ± 2764.6	8269.0 ± 2800.3	4801.5 ± 923.8	5130.3 ± 714.9
AUC_inf_ (ng·h/mL)	4724.20 ± 1093.57	4465.22 ± 1409.58	10959.8 ± 2706.8	8664.0 ± 2961.9	5285.3 ± 1246.1	5814.7 ± 1364.0
t_1/2_ (h)	3.61 ± 1.16	4.88 ± 1.79	3.7 ± 1.2	5.5 ± 2.3	5.6 ± 3.3	7.6 ± 6.0
	**Statistical BE test of F1**		**Statistical BE test of F2**		**Statistical BE test of F3**	
**Bioequivalence**	C_max_ (ng/mL)	AUC_last_ (ng·h/mL)	C_max_ (ng/mL)	AUC_last_ (ng·h/mL)	C_max_ (ng/mL)	AUC_last_ (ng·h/mL)
Geometric mean ratio (Test/Ref, %)	87.93	92.28	75.48	75.46	130.92	107.82
90% CI (Lower)	77.50	79.28	57.93	51.87	105.05	89.32
90% CI (Upper)	99.76	107.42	98.35	109.78	163.17	130.16

* Data are mean ± standard deviation (SD, *n* = 6). ** Median value [Minimum value–Maximum value]. Reference drug, Stablon^®^ tablets (tianeptine sodium immediate-release tablet/taken three times a day); Test drug, TSR tablets (tianeptine sodium sustained-release tablet/taken once a day).

**Table 3:** Formulation of TSR Tablet (*w*/*w*%).

Ingredient	Formulation	
	IR (Immediate-Release)	SR (Sustained-Release)
Tianeptine sodium	4.3 (6.5 mg)	5.2 (31 mg)
MCC ^1^	64.4	-
D-mannitol	26.8	59.2
HPMC ^2^ 100M cp	-	30.0
PEO ^3^	-	3.3
Mg stearate	1.2	2.0
colloidal silicon dioxide	3.3	0.3

^1^ MCC: microcellulose crystalline. ^2^ HPMC: hydroxypropyl Methylcellulose. ^3^ PEO: poly(oxyethylene).

## Data Availability

The data presented in this study are included in this published article.
